# Petroclival meningiomas

**DOI:** 10.3171/2022.1.FOCVID225

**Published:** 2022-04-01

**Authors:** Sebastien Froelich, Ian F. Dunn, A. Samy Youssef, Nicholas C. Bambakidis

**Affiliations:** 1 Department of Neurosurgery, Hôpital Lariboisière, Paris, France;; 2 Department of Neurosurgery, University of Oklahoma Health Sciences Center, Oklahoma City, Oklahoma;; 3 Department of Neurosurgery, University of Colorado School of Medicine, Aurora, Colorado; and; 4 Department of Neurological Surgery, University Hospitals, Cleveland, Ohio

## INTRODUCTION

The petroclival region of the posterolateral skull base continues to be an anatomic challenge for neurosurgeons. This is particularly true when addressing symptomatic meningiomas, as they are heterogeneous in their nature and clinical behavior, with distinct biologies that we are just beginning to understand. The early surgical history was marked by attempts at understanding how to approach this region, with difficult outcomes; the development of transtemporal approaches was a turning point. Radiosurgery, endonasal and endoscopic techniques, and the concept of the tailored management paradigm are a part of the evolving approach to what remains one of the most challenging tumors in neurosurgery. In any event, petroclival meningiomas continue to be best managed at experienced skull base surgery centers with access to multidisciplinary teams consisting of neurosurgeons, otolaryngologists, radiation oncologists, and, increasingly, medical oncologists. This issue of *Neurosurgical Focus: Video* provides several unique case examples meant to illustrate the range of options deployed today in the management of these challenging tumors.

## Disclosures

The authors report no conflict of interest concerning the materials or methods used in this study or the findings specified in this publication.

